# Severe Sepsis and Wet Gangrene Requiring Foot Amputation Caused by an Emerging Human Pathogen – Shewanella algae

**DOI:** 10.7759/cureus.5668

**Published:** 2019-09-16

**Authors:** Zuzana Talbot, Arun Amble, Guesly Delva, Abdulmagid Eddib, Salman Muddassir

**Affiliations:** 1 Internal Medicine, Oak Hill Hospital, Brooksville, USA; 2 Internal Medicine, Oak Hill Hospital, Brookville, USA; 3 Internal Medicine: Critical Care, Oak Hill Hospital, Brooksville, USA; 4 Internal Medicine, Hospital Corporation of America West Florida GME Consortium / Oak Hill Hospital, Brooksville, USA

**Keywords:** shewanella, shewanella algae, skin and soft tissue infection, bacteremia, wet gangrene, hemorrhagic bulla, seafood, sea water

## Abstract

A 69-year-old woman with type 2 diabetes mellitus, peripheral vascular disease, and other comorbidities presented with recurrent syncopal episodes. Cellulitic skin changes in her right lower extremity were noted, as well as a large hemorrhagic bulla on the dorsum of her right foot. Severe sepsis was determined to be the reason for her syncopal episodes. Blood cultures and the bulla aspirate culture were positive for Shewanella algae that was pan-sensitive to antibiotics. Her clinical status was stabilized with a regimen of intravenous fluids and broad-spectrum antibiotics. However, due to the development of right foot gangrene, she underwent debridement and eventually required transmetatarsal open amputation.

## Introduction

Shewanella species (spp.) are cosmopolitan saprophytic Gram-negative, facultatively anaerobic, motile rods that are part of the normal microflora of the marine environment. Although first isolated in early 1930s (initially named Achromobacter putrefaciens) [1], undergoing reclassification due to gene sequencing methods and being renamed [2], this bacterium was not of a great heed for clinical microbiologists until decades later [3, 4]. Of the 62 species of Shewanella, three have been identified as a cause of infection in humans: Shewanella putrefaciens (S. putrefaciens), Shewanella haliotis (S. haliotis) and, most commonly, Shewanella algae (S. algae) [5, 6].

Shewanella causes four major types of infection: (i) septicemia, (ii) skin and soft tissue infections (SSTI), varying from mild cellulitis to severe life-threatening necrotizing fasciitis, (iii) hepatobiliary infections, and (iv) ear infections [6-16]. Risk factors for infection include mainly mucocutaneous abrasions or penetrating traumas with marine exposure or consumption of seafood [5, 10, 14, 17, 18]. The prospect of developing the illness due to S. algae is highest in patients with vascular conditions, diabetes, hepatobiliary disease, malignancy and otherwise immunocompromised [6, 9, 10, 15]. We present a rare case of sepsis due to S. algae, arising from SSTI, in a patient with no known exposure to this pathogen.

## Case presentation

A 69-year-old female with previous history of type 2 diabetes mellitus, sub-optimally controlled (HbA1c 8.6%), peripheral arterial disease, hypertension, hyperlipidemia, morbid obesity, and stage 3 chronic kidney disease, was brought to the emergency room by her family with multiple episodes of passing out. The patient denied any precipitating factors, prodromal symptoms, or post-event phenomena.

On admission, her blood pressure was 78/43 mmHg, heart rate was 70 beats per minute, temperature 34.4˚C, and she was saturating at 100% on ambient air. She was alert, cooperative, and had no signs of injury. Her heart, lung, abdominal and neurological examinations were unremarkable. Peripheral pulses were non-palpable in lower extremities - there was 2+ non-pitting edema with chronic venous stasis changes bilaterally, erythema and edema of the right foot with large black-colored bulla located at dorsum of distal foot (8 x 10 cm). Laboratory showed leukocytosis with WBC of 16.900/µL and elevated creatinine at 7.8 mg/dL (baseline 1.3 mg/dL, GFR 35 mL/min/1.73 m²).

Based on the clinical presentation and laboratory values, sepsis as the etiology of patient’s syncopal episodes was suspected and aggressive intravenous hydration and broad-spectrum antibiotics (renally dosed vancomycin and piperacillin-tazobactam) were initiated. Blood cultures and right foot bulla aspirate were positive for S. algae that was sensitive to all tested antibiotics. Her clinical status was stabilized, and laboratory parameters improved. However, due to the development of right foot gangrene, she underwent surgical debridement and eventually needed transmetatarsal amputation on the eighth day of her hospital stay.

## Discussion

S. algae is an emerging worldwide pathogen that has recently garnered interest in the medical community. It is a Gram-negative, facultative anaerobic, motile rod found in the normal microflora of marine environments. Early detection and diagnosis are essential to achieving good outcomes and ultimate resolution of the infection.

Shewanella infection usually presents as SSTI of the limb. Diverse presentations from gastroenteritis and peritonitis to meningoencephalitis have been reported in the literature [9, 14]. These infections usually occur in immunocompromised individuals that have been exposed to the pathogen. Often, patients who developed SSTIs and bacteremia reported seawater exposure. There have been several isolated reports of SSTI over the years. In 2008, Tsai et al. reported a series of 27 cases of SSTIs caused by Shewanella bacteria [19]. Twenty-two out of 27 reported cases (81.5%) had limb involvement, out of which 14 (n = 22) had associated bacteremia. A retrospective study in 2010 by To et al. identified 29 patients with isolates of Shewanella species in Hong Kong [20]. Only two of them reported seawater exposure. The authors reported transmission route of the remaining cases remained unclear. Given the popularity of seafood in Hong Kong, it was suspected that the bacteria were transmitted orally [20].

Additionally, Liu et al. in 2013 conducted a retrospective study and also reviewed previously published reports [15]. They identified 59 cases including nine at their hospital. They found that 47.4% patients (n = 28) had underlying hepatobiliary disease and were mainly of Asian descent. Eight out of nine patients (88.8%) in their cohort reportedly lived in the area near the coast and regularly consumed seafood. Their data suggested a link between development of Shewanella bacteremia and seafood ingestion [15].

In 2015, an epidemiological study (n = 16) in India investigated clinical characteristics of SSTI caused by Shewanella spp. [10]. The authors reported S. algae was isolated in 12 cases (75%). All study patients had a clear entry port for the infection in their skin or mucosa and 56.3% were in contact with seawater.

Torri et al. in 2017 published a case series of 17 patients with Shewanella infection in Italy [18]. Among these patients, five (29.4%) had a SSTI and another five (29.4%) had sepsis. Furthermore, to support the significance of seawater exposure in the pathogenesis of the Shewanella infection, Tori et al. reported that two patients in the sepsis group and four patients in the SSTI group, respectively, had contact with seawater within previous four weeks. The only patient who died had Shewanella positive blood cultures and sepsis [18].

A high index of suspicion is therefore required to expeditiously diagnose and treat Shewanella infections. Shewanella spp. should be considered in individuals who present with limb infections such as cellulitis, ulcers, or necrotizing wounds. These individuals include those with predisposing factors such as diabetes mellitus, immunocompromised state, and those with seawater exposure. Albeit lack of any of the listed predisposing factors is not a reason to exclude Shewanella infection. Diagnosis is made primarily by wound or blood cultures and can be confirmed with advanced techniques such as polymerase chain reaction (PCR) or immunofluorescence assays. Broad-spectrum antibiotics should be initiated upon suspicion. Review of prior case reports seems to suggest that cefepime is generally effective against Shewanella, though antibiotic resistance is developing [4, 15, 18-20]. In our patient, the bacteria were pan-sensitive to all antibiotics tested in the laboratory.

Delay in diagnosis and treatment could result in severe complications. These complications include septic shock, necrotizing fasciitis, limb amputation, and even death [9, 10, 15, 18-20]. Though early antibiotic treatment was initiated, our patient, unfortunately, developed wet gangrene of her right great toe that ultimately required transmetatarsal amputation.

Multiple comorbid conditions likely predisposed her to developing an illness due to S. algae. Nevertheless, the mode of acquisition of her infection remained unclear, since she denied any activity related to seawater or seafood consumption during past years. Her only “exposure” is living in warm climate near the coastal area of Southwest Florida.

Although we have reports of S. algae infections from around the globe, the vast majority of reported cases are from the Western Pacific or Mediterranean [15, 18, 20]. Only a handful of cases from the United States were found in literature [5, 16]. A major limitation is the fact that we lacked substantial information about the epidemiology of this rare pathogen able to survive in aerobic and anaerobic environment of various temperatures. More data is required to understand the pathogenicity of S. algae, possible routes of transmission, and treatment.

## Conclusions

Although Shewanella infections can be fatal, most patients appear to have good clinical outcomes as long as prompt treatment is initiated, including medical, surgical and supportive measures. We recommend that clinicians include this organism into their differential diagnosis in patients with the above clinical factors. Patients with risk factors should be careful during any activity in proximity to the sea water as well as when consuming seafood.
